# Fluorescence Resonance Energy Transfer Systems in Supramolecular Macrocyclic Chemistry

**DOI:** 10.3390/molecules22101640

**Published:** 2017-09-29

**Authors:** Xin-Yue Lou, Nan Song, Ying-Wei Yang

**Affiliations:** International Joint Research Laboratory of Nano-Micro Architecture Chemistry (NMAC), College of Chemistry, Jilin University, 2699 Qianjin Street, Changchun 130012, China; louxy1995@icloud.com (X.-Y.L.); nsong15@mails.jlu.edu.cn (N.S.)

**Keywords:** calixarene, cucurbituril, cyclodextrin, host-guest chemistry, pillararene, supramolecular chemistry

## Abstract

The fabrication of smart materials is gradually becoming a research focus in nanotechnology and materials science. An important criterion of smart materials is the capacity of stimuli-responsiveness, while another lies in selective recognition. Accordingly, supramolecular host-guest chemistry has proven a promising support for building intelligent, responsive systems; hence, synthetic macrocyclic hosts, such as calixarenes, cucurbiturils, cyclodextrins, and pillararenes, have been used as ideal building blocks. Meanwhile, manipulating and harnessing light artificially is always an intensive attempt for scientists in order to meet the urgent demands of technological developments. Fluorescence resonance energy transfer (FRET), known as a well-studied luminescent activity and also a powerful tool in spectroscopic area, has been investigated from various facets, of which the application range has been broadly expanded. In this review, the innovative collaboration between FRET and supramolecular macrocyclic chemistry will be presented and depicted with typical examples. Facilitated by the dynamic features of supramolecular macrocyclic motifs, a large variety of FRET systems have been designed and organized, resulting in promising optical materials with potential for applications in protein assembly, enzyme assays, diagnosis, drug delivery monitoring, sensing, photosynthesis mimicking and chemical encryption.

## 1. Introduction

Supramolecular chemistry, since formally proposed and demonstrated to be of great significance in the realization of molecular recognition and assembly through weak and reversible noncovalent interactions, has attracted considerable attention in the fields of chemistry and materials science [1]. Artificial complexes with diverse properties and various functions formed via noncovalent forces have been emerging dramatically in recent years [2,3,4]. The fact that the Nobel Prize in chemistry in 2016 was awarded to Sauvage, Stoddart, and Feringa “for the design and synthesis of molecular machines” even further manifested the increasingly recognized importance attached to this newly-explored realm of chemical science [5].

A major branch of supramolecular research lies in macrocycle-based host-guest chemistry. A host-guest system usually consists of two fundamental components—a macrocyclic host and a suitable guest molecule binding to each other and forming an inclusive complex through weak intermolecular forces, such as hydrophobic forces, electrostatic interactions, hydrogen bonding, Van der Waals forces, etc. Additionally, whether the size of the guest matches the cavity of the macrocycle also plays a crucial role [6,7,8,9]. The above-mentioned features endow the matching of a host-guest pair with rational selectivity. In other words, either entity in a host-guest pair would choose and connect to each other selectively and spontaneously, thus realizing molecular recognition [1,7]. On the other hand, since the noncovalent interactions are weak forces, the host-guest interactions possess nice reversibility and responsiveness towards various factors in the ambient environment such as pH, light radiation, redox reactions, competitive factors, chemical signals, and biological interfering, etc. [7,8,9,10]. This “stimuli-responsive” property of host-guest systems plays a vital role in the construction of artificial molecular machines and nanoscaled smart materials [11,12].

**Figure 1:**
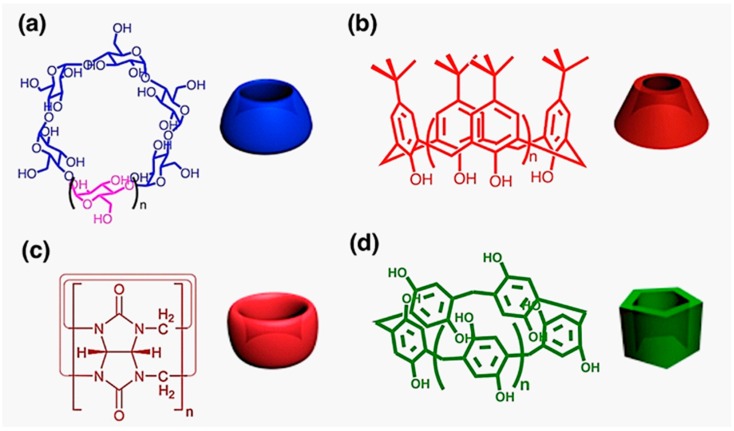
Molecular structures of the four types of supramolecular macrocycles: (**a**) CDs, n = 1–3; (**b**) calixarenes, n = 1–3; (**c**) CB[n]s, n = 5–8, 10, 13–15; (**d**) pillararenes, n = 1–11; and their cartoon depictions.

As any splendid architectures in the world should require elaborately-manufactured building blocks, the fabrication of complicated supramolecular complexes in the nanoscale world can only be achieved when the basic molecular entities are well-designed and synthesized. The characteristics of the macrocyclic hosts, such as structural rigidity, functionalizing possibilities, inclusion properties, etc., perform an essential part in the host-guest activities and their potential applications [7]. Since Charles Petersen first synthesized crown ethers in 1967 and inspired the later development of host-guest chemistry [7], a large amount of macrocyclic compounds have arisen into scientific scope in the subsequent five decades, among which four individuals are receiving significant research concentration, with cyclodextrins (CDs) as the natural macrocycles, the other three, artificial, namely, calixarenes, cucurbiturils (CBs), and pillararenes (Figure 1) [13,14,15,16,17,18,19,20,21,22,23,24,25,26,27,28]. On account of their mature synthetic protocols, comparatively high yields, and versatile chemical modifications, the host-guest properties of the aforementioned four macrocycles are intensely investigated, resulting in the frequent utilization of these supramolecular host molecules [7,29].

CDs are a family of toroid-shaped compounds formed by the cyclization of six or more glucose molecules (with prefixes 𝛼-, β-, 𝛾-, for six, seven, and eight repeating units). Being hydrophilic in the periphery and relatively hydrophobic in the cavity according to their structures, CDs possess unique selectivity on the guest molecules in the formation of host-guest systems. Exploited more than 120 years ago as a natural product, CDs belong to the first generation of macrocycles [13,14].

Calixarenes, as a kind of phenol-formaldehyde macrocyclic oligomers with a cup-like structure, was first discovered from the Bakelite process. Their unique structural skeleton, easy superficial adjustment, and modifiable host-guest properties earned this type of macrocycle sufficient exploration by chemists to widen the relative range of guest molecules. Following crown ethers, calixarenes are the third generation macrocyclic compounds. Their ease of coordination with transition-metal ions is also a key point for exploiting the applications of calixarenes [15,16].

CB[n]s (n = 5–8, 10, 13–15) are synthetic macrocycles with a barrel shape based on repeating monomer elements of glycoluril. With the hydrophobic cavity and polar carbonyl groups on the portals, the CB[n]s family possesses good water-solubility and highly-selective binding interactions with cationic guest molecules driven by several forces including ion-dipole interactions, hydrogen bonding and hydrophobic forces[17,18].

Pillar[n]arenes (n = 5–15), as a newly-synthesized group of macrocyclic hosts consisting of hydroquinone units connected by methylene bridges at 2,5-positions, have been under intense investigation due to their unique structures, controllable host-guest properties and functionalizing capabilities since first reported by Ogoshi et al. Additionally, they have been proved to be good candidates as host molecules for diverse electron-deficient guests and hydrophobic molecules due to their robust electron-rich cavity [19,20,21,22].

Comprehensive reviews have been published covering the macrocycles presented above and have provided sufficient resources for reference, so we will not exhaust the characteristics of these host molecules; instead, we will focus on one of their budding applications that have just been explored in recent years. Just like the phenomenon of mutualism in nature or the synergistic mechanism of enzymes, the realization of multifunction always entails the cooperation of different entities [23]. For instance, to achieve divergent utilities combined with the properties of host-guest chemistry, synthesized macrocyclic hosts are, in most cases, allied to other functional groups to obtain stimuli-responsive smart materials [8,12,24,25]. Existing examples comprise mesoporous silica nanoparticles [24,26] or metal organic frameworks [25] gated by supramolecular switches for drug delivery. In recent periods, increasing attention is being paid to the combination of host-guest pairs and dye molecules to yield various supramolecular systems with unique luminescent properties, such as fluorescence-enhanced supramolecular polymers [27,28], multicolour photoluminescence host-guest complexes [30,31], and so on. One boosting approach of fabricating functional fluorescent supramolecular materials is merging host-guest interactions with fluorescence resonance energy transfer (FRET) effects [32,33] for the purpose of tailoring novel dynamic fluorescent materials with supramolecular properties.

Known as one of the most powerful spectroscopic technologies, FRET remains a focus of interest among researchers since it was first proposed theoretically by Theodor Förster, and then highlighted by the discovery of green fluorescent proteins (GFPs) [34,35]. Since detailed physical mechanisms are beyond the covering region of this review, we give a brief explanation of FRET herein. Resulting from dipole-dipole coupling, a FRET phenomenon involves at least two fluorophores, known as the “donor” and “acceptor”, respectively [34]. To guarantee the occurrence of energy transfer, a sufficient overlap between the emission band of one molecule and the absorption band of the other is always required, which is fundamental to a FRET process, considering the well-studied nature of molecular electronic excitation and energy release [33]. Apart from the emission/absorption spectra, another two factors—the donor-acceptor distance and the relative orientation—also play crucial roles in the fluorescent activities of the FRET pairs [36]. Hence, while in proper proximity, as well as orientation to each other, as the donor molecule is excited by light, the energy released by its excited electrons is transferred to the accepter. As a result, either enhanced luminescent emission of the acceptor is performed visually, or else dark quenching can be expected [37]. Notably, the rate of a FRET effect depends strongly on the distance between the donor and acceptor. Equation (1), promoted by Förster, expresses this dependence nicely:

(1) $$E=\frac{R_{0}^{6}}{R_{0}^{6}+r^{6}}$$

where *E* represents the energy transfer efficiency and *r* stands for the donor-acceptor distance. *R*_0_ is a parameter known as the Förster radius, which varies among different donor-acceptor pairs and depends highly on their spectral overlap. When the donor-acceptor distance is equal to *R*_0_, the transfer efficiency equals 50%. It could be inferred that the detectable FRET efficiency would undergo an apparent shift as the donor-acceptor distance changes and, as a result, fluorescent colour variations are exhibited, which could be observed by naked eyes. Meanwhile, the orientation between the FRET pair also has a large impact on the efficacy of the energy transfer on account of the dipole-dipole interactions amongst the entities [34]. Accordingly, due to the high sensitivity to the donor-acceptor distance alteration and visual characteristics, FRET gained the reputation of “molecular spy” or “spectroscopic ruler” [37].

Generally, three types of fluorescent activities in a FRET process are essentially exploited, namely, the turn-off effect on the donor, the switch-on fluorescence of the acceptor and, sometimes, the quenching of both portions in the cases where the acceptor possesses fluorescence quenching ability. The exploitation of these activities in a FRET process requires controllable changes to occur in the mutual proximity between the donor and the acceptor [34]. However, several major drawbacks exist in the conventional methods of constructing FRET-capable fluorescent materials adopted previously, including the lack of tunability due to the covalent binding manners of the FRET pairs, poor recognizing ability between individual molecules (or functional groups) and low compatibility in water phases or biological systems [33].

Remarkably, the integration of FRET effects with host-guest chemistry, as a promising resolution to this hindrance, successfully furnished the association and dissociation of the donor-acceptor pair with extraordinary flexibility and controllability due to the distinct properties of stimuli-responsiveness and the selectivity possessed by host-guest interactions. This breakthrough has astoundingly inspired a variety of applications of FRET, including real-time in vivo monitoring of biomolecules, such as DNA and proteins, and structural manipulation [38,39,40], cell imaging, drug delivery [41,42], chemical [43,44] and biological sensing [45,46,47], photosynthesis mimicking [48,49], and other relative scientific areas, which we will explore in the following part.

## 2. Basic Studies for FRET Systems Based on Host-Guest Chemistry

The simplest and most fundamental way to build up a FRET system with designed host-guest pairs serving as scaffolds or bridges is constructing dye-containing rotaxanes or mechanically-interlocked molecules (MIMs), in that the earliest studies on the localizing effect induced by host-guest chemistry on the fluorophores required a relatively rigid structure for proper placement. For example, Pei et al. reported a [3]rotaxane complex synthesized via click reactions, constituting two FRET donor groups on the wheels (crown ether) and an acceptor on the axis [50]. Mimicking the natural process of photosynthesis, intramolecular energy transfer was observed from the periphery to the molecular core, attributing to the ideal topology determined by the host-guest pairs. Additionally, they improved this complex by constructing a hyperbranched polyrotaxane with a donor moiety linked to three wheel groups, forming a 2:3 FRET donor-acceptor mode in the multi-rotaxane architecture [51].

**Figure 2:**
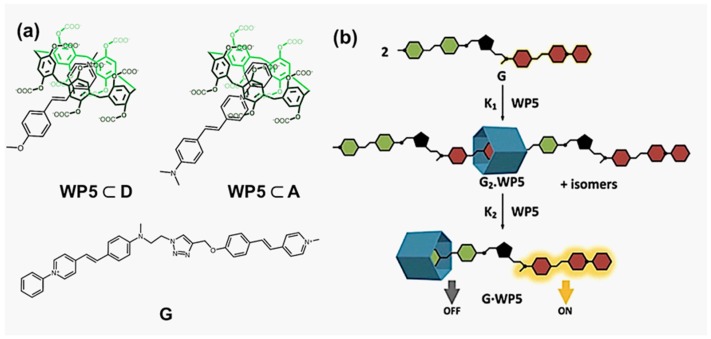
(**a**) The chemical structures of host-guest complexes WP5⊂D, WP5⊂A and G; and (**b**) the cartoon illustration of WP5⊂G [53]. Reproduced by permission of [53]. Copyright 2016 Elsevier.

Subsequently, proceeding research work tempted to penetrate deeper in the scope by exploring the external impacts on the FRET effect imparted by host-guest interactions, with a broader investigating range covering both MIMs and pseudorotaxanes. In 2013, Ogoshi et al. reported the fabrication of a FRET system in a rotaxane complex consisting of a dipyrene functionalized pillar[5]arene (H1) as the wheel and an axle with a perylene stopper (D) [52]. Later in 2016, Bitter and coworkers accomplished the modulation of FRET with water-soluble pillar[5]arenes (WP5) [53]. As depicted in Figure 2, carboxylatopillar[5]arenes were synthesized to serve as the macrocyclic hosts for two dye molecules, presented as D and A, respectively, forming FRET dyad through host-guest interactions. The fluorescent activities were influenced by the formation of inclusion complex due to its impact on the polarity of the guests. The authors, thereafter, synthesized a ditopic guest molecule G by linking D and A through click reaction. The FRET effect was clearly observed in the solution of G. Upon the addition of WP5, there was an intense enhancement of the emission of G(A) moiety, while the fluorescence of G(D) was quenched.

From the studies focusing on the constructing methods for the simple MIMs to intricate supramolecular fluorescent systems, more sophisticated FRET materials controlled by host-guest interactions have been gradually emerging during the past few years in divergent manners, including the fabrication of host-guest complexes, supramolecular polymers, supramolecular nanoparticles and the interposition of inorganic elements. Empirically, in almost every single system involving FRET as a major investigating point, to play tricks with light means either giving out signals or harnessing energy. In cases where sensing, detecting or imaging takes the prior part of the research objective, the success of the materials counts on achieving good distinctness and accuracy of the fluorescent signals. Under this circumstance, apparent variation of the fluorescence is required, which means that the chosen host-guest entities must respond to the stimulus in particular condition so that the efficient functionality could be performed. On the other hand, when the emphasis is placed on the transported energy, in a photosynthesis mimicking system, for example, the goal lies in the high efficacy of the energy transfer process and broadening the adsorption band. This requires the emergence of the host-guest complexes must bring the FRET pairs to the suitable and rigid relative position so as to guarantee the energy transfer efficiency. Apart from the above two types of exploitation of the host-guest components, supramolecular macrocycles, especially CDs, were also introduced into FRET systems to form host-guest pairs, mainly for the purposes of improving the water-solubility and biocompatibility or reducing the toxicity [54,55]. Although these studies comprise both host-guest units and FRET elements, the unique advantages of host-guest chemistry were not demonstrated to the fullest, so we will not include them in the main discussion.

Bearing this in mind, more specific manifestation will be introduced in exact relevance to particular applications in constructing host-guest FRET complexes. In the following part of this review, we’ll provide a comprehensive prospectus on the recent development of FRET materials based on host-guest chemistry. Supreme applications for the novel FRET materials supported by host-guest building blocks will be discussed, including protein assembly [38,39], enzyme assays [56], diagnosis [57], drug delivery monitoring [41,42], sensing [43,44,45,46,47], photosynthesis mimicking [48,49], and chemical encryption materials [58,59] achieved by the collaboration. 

## 3. Applications of FRET Systems Based on Host-Guest Chemistry

### 3.1. Protein Assembly

A vital biological approach involving FRET in host-guest systems is the controlled assembly of biological macromolecules, such as proteins and DNA. In 2010, Brunsveld and coworkers reported the dimerization of monomeric fluorescent proteins induced by the formation of host-guest complex between cucurbit[8]uril and phenylalanine-glycine-glycine (FGG) tripeptide at a ratio of 1:2 [38]. FGG motifs were immobilized on yellow fluorescent protein (YFP) and cyan fluorescent protein (CFP) genetically on the N-termini, which was occupied by methionine, naturally. The host molecule, cucurbit[8]uril, was connected specifically to two FGG groups as the guests in apparent preference to methionine in the controlled experiment, indicating the selectiveness of the host-guest recognition. Homo-FRET (decrease of anisotropy) and hetero-FRET were observed, respectively, when homo- and heterodimers formed, thus, FRET was taken advantage of to visualize the process. The supramolecular inducer of fluorescent-protein-dimerization is the first example of introducing host-guest systems in the activities of proteins, providing a novel insight in manipulating the interactions of biological molecules.

Having demonstrated this new pathway for controlled dimerization of proteins with supramolecular inducers, they continued the research in 2011 by replacing the tripeptides with methoxynaphthol (Np) and methylviologen (MV), which were linked to CFP and YFP, respectively, and would form a charge transfer complex in the cavity of cucurbit[8]uril [39]. In this case, only heterodimers were yielded in the system, and this specific dimerization was envisaged by FRET. Remarkably, the two guest moieties were artificially attached, yielding host-guest complexes and efficiently eliminating unspecified supramolecular interplay with other parts of proteins.

### 3.2. Enzyme Assays

Apart from the manipulation of biomolecular motions, it is also necessary that we cover the investigation on assaying biological functional complexes, such as enzymes. One of the most favourable methods for the detection of enzymes is monitoring the products yielded in the enzymatic reactions.

In a most recent study, a new pathway of enzymatic cleavage assay based on fluorescent dye capture through forming host-guest pairs was developed by Smith and coworkers [56]. Viral neuraminidase (VNA), as an indicator of influenza, was detected taking advantage of its enzymatic cleavage reactions. A squaraine (a dye molecule)-derived compound with two blocking groups at both end was synthesized as the enzymatic substrate while a tetralactam macrocycle with anthracene sidewalls (M) functioned as the supramolecular capture agent. Once the blocking groups were removed by enzyme cleavage, the fragment left of the substrate was threaded into the macrocycle cavity through hydrogen bonding and hydrophobic stacking, conceptually forming a structure of a [2]pseudrotaxane. Placed at an appropriate proximity, FRET occurred from the anthracene group to the squaraine core, resulting in strong fluorescent signals. Meanwhile, affinity capture beads were utilized as a heterogeneous capture assay agent with M functionalized on the surface. Furthermore, various VNA inhibitor drugs were tested, whose efficiency was examined according to their FRET signals.

### 3.3. Diagnosis

**Figure 3:**
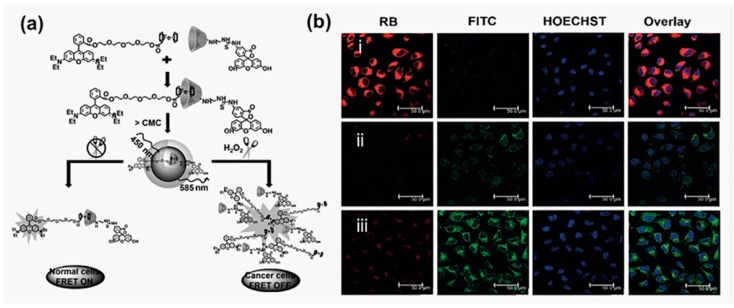
(**a**) Nanoparticles assembled by amphiphilic host-guest pairs in the “FRET-on” state responsive to the ferrocene reduction by ambient H_2_O_2_; (**b**) Confocal fluorescence images of HeLa and L929 cells with (i) L929 cells treated with 5 µm FITC-β-CD/Fc-RB; (ii) L929 cells incubated with 50 µm H_2_O_2_ and 5 µm FITC-β-CD/Fc-RB for 4 h and (iii) HeLa cells incubated with 5 µm FITC-β-CD/Fc-RB for 1 h at 37 °C [57]. Reprinted with permission from [57]. Copyright 2015 Wiley.

In 2015, Wang and coworkers reported the supramolecular fluorescent nanoparticles tailored for the detection of hydrogen peroxide (H_2_O_2_) in cancer cells by means of FRET [57], which is depicted in Figure 3a. Fluorescein isothiocyanate (FRET donor) modified β-CD (FITC-β-CD) and rhodamine B (FRET acceptor) modified ferrocene (Fc-RB) were synthesized to produce amphyphilic host-guest pairs linked via the host-guest interaction of β-CD and ferrocene, which spontaneously assembled into nanoparticles in aqueous solution. The key point of H_2_O_2_ detection lay in the mechanism that the ferrocene unit was hydrophobic in its reduction state and hydrophilic in the oxidation state. Consequently, degradation of the nanoparticles took place with the presence of H_2_O_2_ in high concentrations like in cancer cell cytoplasm, according to the incompatible inclusion of oxidized ferrocene in the hydrophobic cavity of β-CD. The fluorescence colour was also turned from red to green along with the separation of the FRET pairs. In comparison, the emission remained green in L929 cells due to the lack of H_2_O_2_. The responsiveness toward H_2_O_2_ concentration provided a visible signal of the existence of concentrated H_2_O_2_ and also a powerful evidence for distinguishing normal tissues and cancer cells (Figure 3b).

### 3.4. Drug Delivery Monitoring

Nanocarriers have been tailored for the delivery and controlled release of drugs and biological materials for years. Yet the cargo loading and release of commonly used carriers are hard to monitor, especially under in vivo circumstances. In 2016, Huskens and coworkers reported supramolecular nanoparticles (SNPs) labelled by FRET pairs for drug delivery and responsive imaging taking advantage of the electrostatic forces between the oppositely-charged carriers and cargos, while the charged SNPs were stabilized by the CD though host-guest interactions [60].

Huang and coworkers reported a [2]rotaxane complex with pillar[5]arene (P5) for mitochondria imaging and drug delivery materials [41]. In this study, concepts of aggregation-induced emission (AIE) and aggregation-caused quenching (ACQ) were both introduced in host-guest controlled FRET study. By anchoring a tetraphenylethylene (TPE) unit, a typical AIE-active luminophore, as a stopper on one end of the axle and pillar[5]arene serving as the wheel, a [2]rotaxane was formed with enhanced fluorescence according to the AIE effect. The anticancer drug, Doxorubicin (DOX), and the ACQ agent as well, was covalently linked to the wheel via imine bridges and, thus, was placed close to TPE, leading to dramatic failing of the fluorescent emission due to the ACQ effect, which occurred upon FRET from TPE to DOX. The “dual-fluorescence quenched” complexes later self-assembled into nanoparticles, which could undergo hydrolysis in endo/lysosomes and the breakage of the imine groups, releasing DOX in the cytoplasm. Due to the extra negative membrane potential of mitochondria, it can be recognized by the [2]rotaxane through electrostatic interactions and be lighted up through the recognition.

**Figure 4:**
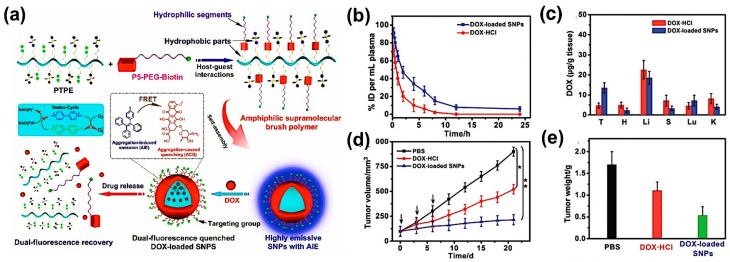
(**a**) Procedure of the formation and disassembly of SNPs with DOX loading and dual fluorescence quenching; (**b**–**e**) Graphical demonstration for the in vivo effects of the drug-delivering SNPs: (**b**) blood circulation time of DOX·HCl and DOX-loaded SNPs analyzed through the plasma concentration of DOX after injection; (**c**) Distributions among the main organs (tumour, heart, liver, spleen, lung and kidney) of DOX at 12 h post-injection; (**d**) Tumour growth inhibition curves on the HeLa tumour model treated by phosphate buffered saline(PBS), DOX·HCl and DOX-loaded SNPs respectively; (**e**) The average weight of the tumours of mice bearing HeLa tumours after the aforementioned three different treatments [42]. Copyright 2016. Reprinted with permission of The Royal Society of Chemistry.

With this foundation, they continued to fabricate FRET-capable SNPs for DOX delivery assembled by pillar[5]arene-based amphyphilic supramolecular brush copolymers (SBPs) [42]. TPE and 4,4′-bipyridinium derivative (M) moieties were grafted onto the polymer backbone lternately. As shown in Figure 4a, host-guest interactions formed between the M entities and PEG-Biotin (targeting group) functionalized P5. Hence, SBPs were constructed and subsequently self-assembled into SNPs, which exhibited an AIE effect originating from the aggregation of TPE units in the core of the particles. Once DOX was encapsulated into the SNPs, the emission of the system declined because of the FRET from TPE to DOX and the ACQ of the DOX units, resulting in dual-fluorescence quenching. When the guest M was reduced by the intracellular reductase NAD(P)H in an acidic environment from a bicationic entity to its radical cationic state, the binding between them and P5 were intensely weakened, with the association constant dwindling by two orders of magnitude, leading to the detachment of the host-guest pair and the disassociation of the SNPs. In this way, DOX was released from the confinement of the particles, and the fluorescence recovered as well. The controlled drug releasing properties, the distributions in normal tissues and cancer cell inhibition efficiency were investigated as shown in Figure 4b–e, demonstrating the nice encapsulation and cancer targeting capacity (DOX concentration was higher in tumour treated with DOX-loaded SNPs than with DOX alone in contrast to that in other organs). Uniquely, in this study, the proximity/separation of the FRET pair was not controlled by host-guest interactions directly, but in a rather circuitous, yet subtle, way by manipulating the assembly of SBPs and FRET at the same time, performing an elegant cooperation between stimuli-responsiveness of host-guest chemistry and fluorescent signalling of FRET.

### 3.5. Sensing

The introduction of host-guest chemistry into FRET systems has also inspired relevant studies aiming at tailoring novel chemosensors and biosensors with enhanced properties by offering either rigid molecular scaffolds or selective binding sites for the sensing functionalities. Therefore, it is necessary for us to introduce FRET effects based on host-guest interactions recruited in the fields of nano-sensing with the latest research achievements exhibited.

Among the massive group of fluorescent chemosensors reported in the last few decades, an important impact is the detection of deleterious matter, such as heavy/transition metal ions that are harmful to the environment and human health [61]. According to previous literature, the utilization of tailored macrocycles, especially calix[n]arenes, has already emerged in chemical sensing scope in several cases benefitting from the desired rigidity, changeable conformations, and various possibilities of functionalization of the asymmetric cyclic structure [15]. For instance, calix[4]crown, with the functionality of double-recognition, was covalently linked to pyrene by the lower rims to form a switchable excimer-based chemosensor for lead ions in the work by Kim and coworkers in 2004 [58]. Later, in 2007 and 2010, another two chemosensors were tailored successively for mercury ions [62,63]. Differently, instead of an excimer, FRET acted as the sensing signals in these two systems. The conformational alterations induced by coordination between ions and the recognition units played a crucial part in the sensing process. With these inspirational predecessors, the combination of FRET and host-guest recognition got employed in this research realm in the last few years and proved an effective pathway for sensing detrimental ions.

In 2009, Yu and coworkers reported a FRET-based approach to detect ferric ions ratiometrically via a water-soluble host-guest complex containing donor (dansyl group)-linked β-CD and acceptor (spirolactam rhodamine)-linked adamantine [43]. A ring-opening reaction was induced by ferric ions efficiently in the acceptor by coordination, reforming the moiety into a longer-wavelength fluorophore comparing to the dansyl part, hence, switching on the FRET. The quantity of Fe^3+^ could be calculated according to the emission spectra. The host-guest complex not only retained the donor and acceptor within their effective distance, but also avoided the unwanted interactions between the two portions, guaranteeing the structural intactness. Similarly, benefitting from the analogous mechanism, Lü and coworkers developed a ratiometric Hg^2+^ sensor by grafting FRET-donor-containing conjugated polymers on the surface of mesoporous silica nanoparticles (MSNs) as a robust solid scaffold, on which β-CDs were anchored and then the acceptor predecessor groups were attached through host-guest recognition [44]. Fluorescent signals were observed upon the ring-opening effect of Hg^2+^ on the acceptor precursor. Moreover, this newly-designed sensor was proven capable of detecting Hg^2+^ in preference to other metal ions, showing a brilliant advantage in selectivity.

**Figure 5:**
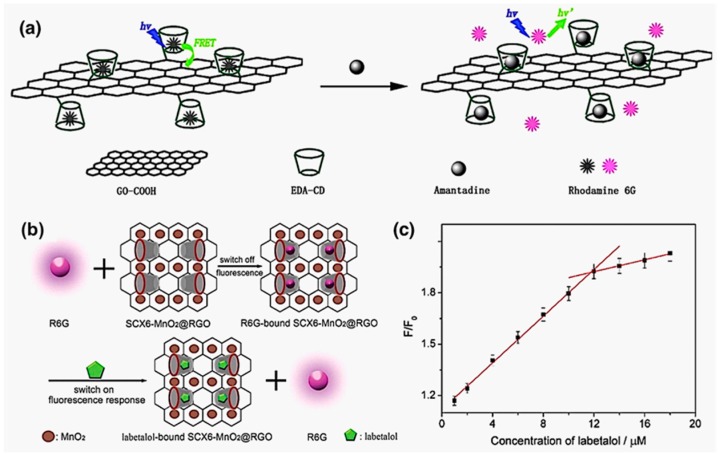
Illustrations for (**a**) the sensing of amantadine by the competitive binding against R6G and (**b**) the sensing of labetalol by the competitive binding against R6G, accompanied by fluorescence recovery of the freed R6G; (**c**) Calibration curves of fluorescence intensity of R6G•SCX6-MnO_2_@RGO proportional to added labetalol concentration [46,47]. Copyright 2014 and 2016. Reprinted with permission of Elsevier and The Royal Society of Chemistry.

While the sensing of hazardous ions tends to take advantage of coordination-induced conformational changes to trigger FRET signals, the detection of electroneutral composites, such as biological compounds, are commonly achieved in an approach of competitive binding. Early in 2003, there was already research work reported which employed FRET controlled by competitive host-guest interactions as a fluorescent probe for conformational studies of helical peptides in aqueous solution [40]. This stratagem was later used in pharmaceutical or metabolic detections. For example, in 2014, Su and coworkers reported the construction of an amantadine sensor based on competitive host-guest interactions and a FRET-quenching platform of graphene oxide (GO) [46]. As shown in Figure 5a, β-CDs were immobilized on the graphene oxide sheet covalently as the host moieties. Well fitting for the inclusion of β-CDs, rhodamine 6G (R6G) served as the fluorescent probe to compete with the sensing target—amantadine—which could form more stable host-guest pairs with the identical hosts. Hence, apparent quenching of fluorescence was observed in the system in the absence of amantadine according to the FRET from R6G to GO, yet once amantadine was introduced, releasing R6G from the GO-CD complex, the fluorescence recovered immediately in a mode linearly relational to the amount of added amantadine.

Subsequently, Xie et al. published their work on the development of the FRET sensor for labetalol (heart-rate reducer) following the same principle as above. [47] *p*-Sulfonated calix[6]arene (SCX6) was selected to be the host molecule and Rhodamine 6G (R6G) as the competitive agent (Figure 5b). However, they made a remarkable difference by doping MnO_2_ on the reduced graphene oxide monosheet to yield MnO_2_@RGO—a fluorescence quencher with higher efficiency. They further explored on the binding properties of labetalol and R6G with SCX6, coming to a conclusion that labetalol could bind much stronger to SCX6 than R6G, which exactly agreed with the FRET signals of competitive binding. The intensity of the recovered fluorescence was found proportional to the amount of the labetalol added, as depicted in Figure 5c, thus, ratiometric analysis was achieved.

Another significant application of biosensing is the detection and assessment of metabolites in living organisms. Based upon supramolecular interactions and FRET, Heath and coworkers successfully developed a competitive assay for cellular glutamine (an important metabolite in cancer cells) uptake [45]. Cy3-labeled CDs linked to a single-stranded DNA were immobilized through DNA hybridization on a glass surface modified by the complement sequence, serving as the host and the FRET donor. Then, a dark quencher molecule—BHQ2—and a glutamine analogue were conjugated to an adamantane group, the guest, respectively. While the labelled glutamine analogue was proven capable of being absorbed by cancer cells and selected in a small library of resembling analogues. When adamantane-BHQ2 solution was added to the assay surface, FRET from the Cy3 group to the BHQ2 was triggered, along with the expected fluorescence quenching. Yet the following the introduction of the adamantane-labeled glutamine analogues into the system, on the contrary, retained the fluorescence by way of competing with quenchers over the β-CD hosts since no FRET occurred between Cy3 and the glutamine analogue.

### 3.6. Photosynthesis Mimicking

As nature provides us an ideal prototype for converting solar energy into chemical energy in the form of photosynthesis, artificial light harvesting materials have been advancing for a long time, mimicking the antenna structure for the realization of energy transfer. FRET, as an important step in the whole procedure of photosynthesis, undoubtedly plays an important part in the design and operation of synthesized light harvesting systems. Furthermore, considering the fact that the energy transferring moieties in nature, namely, the FRET donor and acceptor in the antenna, are commonly held in proximity through noncovalent interactions, it can be expected that the introduction of host-guest chemistry would bring evolving inspiration for organic or hybrid materials to mimic the energy transfer process of photosynthesis. 

In the earlier work of MIM-supported FRET systems mentioned in the introduction, the insertion of inclusion effects by designing rotaxane structures effectually promoted the light-harvesting properties through controlling the donor-acceptor position and suppressing chromophore stacking, making a promising archetype for molecular energy transducting devices [50,51]. However, the fixed conformations of MIMs, though providing the FRET pairs with suitable placement, can be a hindrance for flexible manipulation of the host-guest light harvesting systems. Instead, the construction of a pseudorotaxane would lead to large enhancement in the properties of stimuli-responsiveness through attachment and detachment between host and guest moieties.

As a further step for more complicated materials mimicking the energy transferring process, Wang, Li, and coworkers fabricated a FRET based supramolecular polymer. Two guest molecules with two and three linker entities respectively connected to a boron-dipyrromethene (BODIPY) derivative and a ditopic BODIPY host (H) with two pillarenes on both arms were synthesized. Thus, supramolecular polymers were formed in two binding modes—AA/BB-type and A_2_B_3_-type. Both the polymers exhibited broad absorption bands of UV/visual light and FRET efficiency as high as 51% and 63%, respectively, demonstrating the potential for photosynthesis imitation. With the formation of the supramolecular polymer, host-guest interactions offered proper placement and also avoided the unexpected alteration in the donor-acceptor distance, providing a powerful support for the energy transfer.

### 3.7. Chemical Encryption

Apart from harvesting solar light and broadening the adsorption spectra of the materials, another significant actualization of manipulating light is the fabrication of photoluminescent materials whose fluorescent colours can be well-tuned as a promising tool for the utilizations, such as security encryption [58] and signal transduction [59].

**Figure 6:**
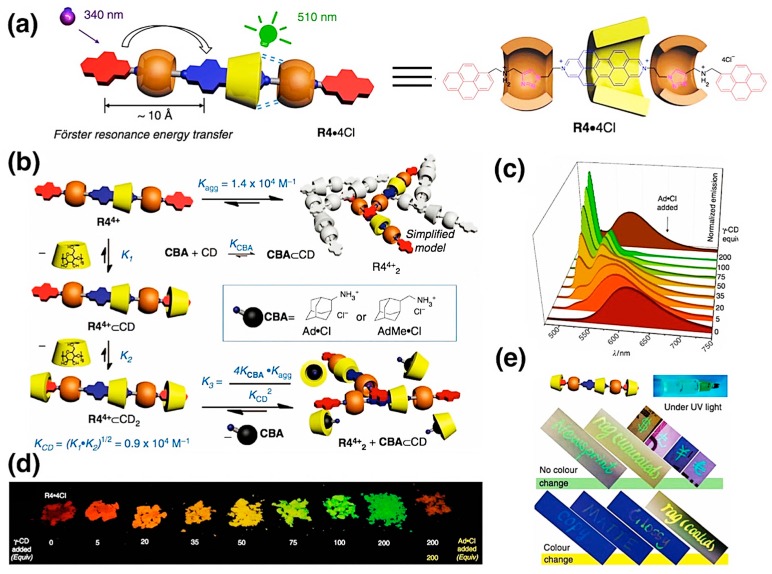
(**a**) Chemical structure of FRET-capable [3]heterorotaxane; (**b**) Schematic illustration of the equilibria involving R4•4Cl as its Cl^−^ salt in the presence of γ-CD and CBAs; (**c**) Solid-state fluorescence spectra (λ_ex_ = 347 nm) of R4•4Cl upon adding 0–200 equiv. of γ-CD, followed by 200 equiv. of Ad•Cl as the CBA; (**d**) Powders obtained from homogeneous mixtures of R4•4Cl and ascending amounts (0–200 equiv) of γ-CD and Ad•Cl (200 equiv) under UV light; (**e**) Surface-dependent fluorescence ink on different paper media (newsprint, coated and uncoated rag paper, banknotes, copy, matte and glossy white paper) under UV light. Copyright 2015 Nature. Reproduced with permission from [58].

In 2015, Stoddart and coworkers reported the solid-state fluorescent materials originated from a FRET-capable heterorotaxane (R4•4Cl) with adjustable fluorescent emissions, which is reversibly manipulated by the supramolecular-controlled aggregation [58] as shown in Figure 6a–b. The basic building motif comprises a FRET pair on the rotaxane axle, bringing about a larger adsorption range, and three macrocyclic wheels threaded on it. The formation of excimers or exciplexes between the rotaxanes through π-π interactions led to the aggregation upon increased concentration, the decline of the fluorescent emission at 510 nm and the emergence of a broader emission band at 610 nm. Based on this fact, 𝛾-CD was introduced into the system for the encapsulation of the pyrene groups, and 2-adamantylamine hydrochloride (Ad•Cl) as competitive binding agents (CBA). Upon adding 𝛾-CD, the congregation was blocked due to the inclusion effect, destroying the excimers or exciplexes, hence, gradually retrieving the original fluorescence as shown in Figure 6c–d. Yet the addition of Ad•Cl, on the contrary, recovered the aggregation state of the materials, thus, forming a dynamic equilibrium. A wide range of colours of fluorescence from red to green was obtained in the process of tuning the assembly by changing the ratio of R4•4Cl, 𝛾-CD, and Ad•Cl. The solid-state materials were utilised as fluorescent ink with 𝛾-CD as the tonor and Ad•Cl (CBA) as the eraser, providing a large colour assay, which could be coded through a non-linear equation. Interestingly, the hybrid ink exhibited different colours when written on different types of paper, possibly resulting from the noncovalent bonding with the different compositions of the papers (Figure 6c–e). Taking advantage of all these dynamic fluorescent activities controlled precisely by supramolecular equilibria, this novel supramolecular fluorescent material possesses large potential to be applied for encryption coding that could neither be tampered nor counterfeited.

Another interesting work lies in the molecular logic gate with FRET as a real-time luminescent responding mediator. Das and coworkers demonstrated the FRET-facilitated supramolecular assembly with operational logic functions on solid surface in 2016 [59]. Self-sorting took place by means of molecular recognition between the chosen host molecules (crown ether derivatives) immobilized on the silica surface and different guests as the inputs, with FRET donor and acceptor groups functionalized on them, respectively. Upon the assembly and disassembly of the host-guest pairs, fluorescent signals would be produced. Thus, through the self-sorting process, logic operators including YES, INHIBIT, OR, and AND were all achieved with their corresponding luminescent responses.

## 4. Conclusions and Perspectives

As a conclusion, the recent developments of FRET host-guest systems for responsive materials and the relative construction strategies have been outlined in this review. The highlighted utilization of four types of supramolecular host molecules, consisting of the natural product CDs and three artificial macrocycles, i.e., calixarenes, CBs, and pillararenes, has exhibited great potential in a bunch of applications including protein assembly, enzyme assays, diagnosis, drug delivery monitoring, sensing, photosynthesis mimicking, and chemical encryption, evincing the excellent feasibility for supramolecular elements, especially the synthesized macrocyclic molecules, to serve as the fundamental building blocks for the constructions of responsive fluorescent materials.

In the final part of this review, it is necessary to make a summary of the divergent constructing modes of host-guest based FRET materials for various applications. When the application targets the pharmaceutical regime, especially for in vivo imaging drug delivery, SNPs have been an excellent choice due to their cargo-encapsulating capacities, fine biocompatibility, and degradability. With the FRET agents offering the optical signals for the loading and releasing processes of the cargo molecules, the tailored fluorescent SNPs are able to provide a multifunctional platform for both cargo delivery and real-time monitoring for the delivering activities instantaneously. However, for diagnostic issues, the constructed systems always contain active functional groups that would undergo conformational or electrical changes when triggered by particular external stimuli, leading to either the assembly/disassembly of the supramolecular complex or the occurrence/expiration of the FRET process, and thus resulting in fluorescent indications for the diagnostic targets. In the sensing part, intramolecular isomerization and intermolecular competitive binding play a crucial role in the effectuation of FRET-supported supramolecular sensors by facilitating the responsive FRET signs according to the presence or absence of the analytes. Additionally, for biosensing issues, an important feature is that the host molecules, as the recognition units, are frequently anchored onto surfaces either as the receptor of the transferred energy or for solid support. For photosynthesis mimicking materials, an ideal approach is fabricating supramolecular polymers with FRET members threaded in the structure. Moreover, taking advantage of the controlled assembly-disassembly events, or the stimuli-responsiveness of host-guest interactions, the mutual distance and orientation of FRET pairs are rendered to be tunable, proving host-guest interactions a powerful tool for altering colours of the emitted lights. For another, an obvious benefit is that the polymers can undergo gelation, hence, making desired solid-state smart fluorescent materials. The combination of host-guest interactions with other optical nanostructures, such as quantum dots, is also a good pathway. As for chemical encryption, the accuracy and diversity of the fluorescent outputs induced by the switchable dynamic equilibrium is important for efficient secrecy or signal transduction, so a multicomponent system with controllable fluorescence variation and a broad range of fluorescent colours is highly required.

In spite of the fact that the innovative cooperation between FRET effects and host-guest chemistry has inspired the fabrication of a variety of smart fluorescent materials with stimuli-responsive properties, there remain many challenges in this upcoming field for researchers to deal with in order to play better tricks with light. We will list four main aspects requiring further improvements in the supramolecular FRET-capable materials which have already been constructed up till today: (i) For the photosynthesis mimicking materials, the solidation is comparatively difficult to realize, or the solid state would turn out to be fragile and susceptible to diverse influences in the ambient environment, owing to the reversible nature of host-guest interactions; (ii) In the biological applications, the multiple factors in vivo should be taken into consideration more excessively. Instead of utilizing artificial stimulating agents, some well-studied reactions in the living organism can be exploited directly as switches, making the responsive FRET systems more particular to different biological conditions; (iii) The introduction of nanotechnology into the investigating range would doubtlessly open up infinite possibilities for the birth of novel material types and unexpected functionalities for many other optical applications, such as information storage or lab-on-a-chip technology. The combination with a large scope of other nanoscaled materials also remains to be realized [64,65,66,67]; (iv) The design of supramolecular FRET-capable materials would surely benefit from emulating natural systems that have been taken advantage of artificially, such as DNA nanomachines. For example, a series of synthesized DNA supramolecular nanostructures have already been reported by Willner and coworkers, which include DNA catenane rotary motor, interlocked DNA Olympiadane, DNAzyme-modified MSNs [68,69,70,71,72]. They recruited FRET effects to track the dynamic features of nanomachines, either for the reconfiguration of the DNA motors or the programmed synthetic processes occurring in the pores of DNAzyme gated MSNs. The advances of these DNA nanomaterials with fluorescence properties might provide inspirations for the constructions of novel supramolecular fluorescent materials based on synthetic macrocycles.

The remarkable development of the synergistically-designed FRET systems supported by supramolecular macrocyclic chemistry has led to the birth of a variety of smart fluorescent materials. We envision that better tricks with light will be played as bolder innovations should be made, so that more elaborately-designed supramolecular FRET-capable fluorescent systems with tailored functionalities will emerge in the future.
